# Rapidly mining candidate cotton drought resistance genes based on key indicators of drought resistance

**DOI:** 10.1186/s12870-024-04801-6

**Published:** 2024-02-21

**Authors:** Shiwei Geng, Wenju Gao, Shengmei Li, Qin Chen, Yang Jiao, Jieyin Zhao, Yuxiang Wang, TingWei Wang, Yanying Qu, Quanjia Chen

**Affiliations:** 1Engineering Research Centre of Cotton, Ministry of Education/College of Agriculture, Xinjiang Agricultural University, 311 Nongda East Road, Urumqi, 830052 China

**Keywords:** *G. hirsutum*, Drought resistance indicators, BSA-seq, Functional verification

## Abstract

**Background:**

Focusing on key indicators of drought resistance is highly important for quickly mining candidate genes related to drought resistance in cotton.

**Results:**

In the present study, drought resistance was identified in drought resistance-related RIL populations during the flowering and boll stages, and multiple traits were evaluated; these traits included three key indicators: plant height (PH), single boll weight (SBW) and transpiration rate (Tr). Based on these three key indicators, three groups of extreme mixing pools were constructed for BSA-seq. Based on the mapping interval of each trait, a total of 6.27 Mb QTL intervals were selected on chromosomes A13 (3.2 Mb), A10 (2.45 Mb) and A07 (0.62 Mb) as the focus of this study. Based on the annotation information and qRT‒PCR analysis, three key genes that may be involved in the drought stress response of cotton were screened: *GhF6'H1*, *Gh3AT1* and *GhPER55*. qRT‒PCR analysis of parental and extreme germplasm materials revealed that the expression of these genes changed significantly under drought stress. Cotton VIGS experiments verified the important impact of key genes on cotton drought resistance.

**Conclusions:**

This study focused on the key indicators of drought resistance, laying the foundation for the rapid mining of drought-resistant candidate genes in cotton and providing genetic resources for directed molecular breeding of drought resistance in cotton.

**Supplementary Information:**

The online version contains supplementary material available at 10.1186/s12870-024-04801-6.

## Background

Drought is one of the most harmful abiotic stresses in agricultural production. Understanding the drought resistance mechanism of crops is highly important for improving the ability of crops to respond to drought stress. Cotton is an economic crop, and its growth and production are strongly affected by drought stress [1]. Under drought stress, cotton plants undergo a series of responses. These responses are part of a comprehensive response produced by the interaction of various factors and are mainly manifested in root development, stomatal closure, photosynthesis reduction, hormone production and active oxygen removal [2–5]. These reactions can strongly hinder the morphological growth and physiological activities of cotton plants.

The rapid development of modern technology has greatly promoted research on the response mechanism of cotton plants to drought stress. Mining more cotton drought resistance genes has greatly contributed to elucidating the underlying molecular mechanism involved. Compared with map-based QTL mapping and GWAS, bulked segregant analysis sequencing (BSA-seq) is considered a low-cost, high-efficiency and rapid QTL detection method. BSA-seq is mainly used to sequence individuals with extreme traits in hybrid families for gene mapping and has been successfully used in rice, sorghum, sunflower, chickpea, peanut, wheat, cotton and other crops. Using the BSA method, Barik et al. [6] genotyped an RIL population and found a new QTL in the population that controlled the content of chlorophyll a during stress periods. Gao et al. [7] used BSA-Seq and RNA-Seq to locate QTLs related to millet dwarfing, revealing the molecular pathways and related genes responsible for millet height. Nguyen et al. [8] used BSA-derived AFLP markers for drought tolerance QTL identification in rice.

For a long time, the complexity of the drought resistance-related traits of cotton has increased the difficulty of evaluating the drought resistance of large cotton populations and mining key genes for drought resistance. Zhang et al. [9] identified six QTL intervals associated with plant height, fresh shoot weight, and fresh root weight in cotton plants under normal control and PEG-simulated drought stress conditions in a greenhouse. Abdelraheem et al. [10] used a multiparent high-generation hybrid population composed of 550 recombinant inbred lines and 11 upland cotton parents based on the two indicators of plant height and dry weight to correlate the drought tolerance of cotton seedlings, and 13 and 7 QTLs were detected, respectively. Guo et al. [11] investigated 189 upland cotton resource materials to obtain phenotypic data for 8 drought-related traits in four environments and used genomic data to carry out association analyses of 5 models. Li et al. [12] used 517 natural populations of upland cotton plants as materials to conduct generalized heritability and coefficient of variation analyses of 18 traits and identified 33 QTLs related to the water limitation response by using the drought resistance coefficient. Although these studies subsequently identified the drought-related functional genes of the corresponding indicators, a single trait indicator may not represent the overall drought resistance of cotton, and selecting multiple trait indicators creates an enormous workload when evaluating large groups.

The establishment of a main effect trait index can reduce the difficulty in identifying drought resistance and mining functional genes from large populations. Sun et al. [13] screened 5 effective cotton drought resistance-related indicators from 19 indicators related to morphology, photosynthesis, physiology and yield via the principal component dimensionality reduction method and evaluated the drought resistance of 104 cotton varieties. With respect to cotton yield traits under drought stress [14], statistical methods have been used to verify that the geometric mean productivity, average productivity and drought resistance index are significantly positively correlated with yield under drought stress and can be used to distinguish cotton plants with high drought tolerance. In addition, Munir et al. [15] combined principal component analysis (PCA) and multivariate linkage analysis to screen for morphological parameters related to an increase in cotton yield and evaluated the level of genetic diversity in different cotton genotypes. Therefore, to support genetic improvement, this study focuses on many traits by using statistical methods and screens out key indicators that can be used as selection criteria for subsequent functional gene mining to improve efficiency when mining key genes involved in drought resistance in cotton to lay a foundation for future work.

## Methods

### Plant material

The materials tested in this study included the American Aizi cotton variety Acala1517-08, which has strong drought resistance, as the male parent, and the drought-sensitive material Xinluzhong 36 (XLZ36), which was the female parent. An RIL with 240 offspring was constructed by the single-seed transmission method. In Kuitun city, Xinjiang, China (44°25′11″N, 84°54′04″E), in 2019–2020, at the Experimental Base of Xinjiang Agricultural University of the 144th Regiment in Shawan City, Xinjiang, China (43°29’– 45°20’N, 84°57’–86°09’E), field planting and data surveys were carried out at a total of 3 environmental points, hereinafter referred to as 19KT, 19SW, and 20SW. The highly drought-resistant Tashkent 7 (TSG7) and the weakly drought-resistant Xinluzao 26 (XLZ26) [16] identified in the resource population by our research group are preserved by the Key Laboratory of Agricultural Biotechnology of Xinjiang Agricultural University.

### Drought stress test at the flowering and boll stage

The flowering and boll stages are when cotton plants transition from reproductive growth to vegetative growth, and this is the growth period during which water has the greatest impact on cotton plants [17]. The drought stress experimental design included areas of normal irrigation (CK) and drought stress treatment (DS), and both treatments were repeated twice randomly within the study area. Drip irrigation under plastic film was adopted for the planted materials, and artificial water control and drought treatment tests were carried out at the flowering and boll stages to simulate water stress. In addition, shortly before the end of the drought stress treatment, a soil sampler was used to collect soil samples at 20 cm, 40 cm, and 60 cm from the normal irrigation and drought stress treatment areas using the 5-point method to calculate the soil moisture content.

### Determination of the photosynthetic indices

A reduction in the photosynthetic index is the main physiological response of cotton plants to drought stress [18]. After the drought stress treatment was completed, the colony material was measured using a Lufthansa 3 portable photosynthetic instrument (CIRAS-3 UK) in our laboratory before rewatering. Five cotton plants with uniform growth and 3 continuous true leaves were selected as the standard. Materials from the normal irrigation treatment and drought stress treatment were measured, and the measurements included the net photosynthetic rate (photosynthetic net, Pn), stomatal conductance (gas cavity, Gs), and six photosynthesis-related indicators, such as the intercellular carbon dioxide concentration (Ci), transpiration rate (transpiration rate, Tr), water use efficiency (WUE) and water pressure deficit (vapor pressure deficit, VPD). Each treatment was repeated twice.

### Determination of agronomic traits and yield traits

On the day the drought stress treatment was completed, the biomass of each material was measured, and the aboveground dry matter accumulation (overground part [OP]) and the root–shoot ratio (root–shoot ratio [RSR]) of the RIL population under the normal irrigation treatment and the drought stress treatment were calculated. The agronomic traits of each type of material in the RIL population were investigated at the mature boll-opening stage by collecting plants with uniform growth and 5 consecutive leaves. The traits measured included plant height (PH), fruit branch number (FBN), effective fruit branch number (EFBN), boll number (BN), and effective boll number (EBN). Furthermore, a total of 20 cotton bolls were collected from the upper, middle and lower fruiting branches of each plant, and the cotton seed yield (CSY), lint yield (CLY), lint percentage (LP), single boll weight (SBW) and other traits were measured.

### Data processing and analysis

The data were collated and summarized in Excel 2010 software, and SPSS 21.0 and RStudio were used to analyze the multienvironment phenotypic point data, including group descriptive statistics, correlation analysis, principal component analysis (PCA) and multienvironment population data analysis. Analytical methods such as cluster analysis focus on the key traits of drought resistance. The drought resistance of the RIL populations was comprehensively evaluated by using the comprehensive drought resistance measurement value D and the relative change rate (V) of traits after stress and supplemented by indicators such as the drought resistance coefficient (DC), drought resistance index (DI), and drought resistance membership function (DM) [13]. TBtools [19] was used to perform cluster mapping on the results of drought resistance classification of the RIL populations.

### BSA-seq sequencing

Based on the relative rate of change after drought stress as a standard, extreme materials with stable performance of key drought resistance traits in multiple environments were screened, and three groups of mixed pools were constructed, namely, the PH pool (25 drought-resistant + 25 drought-sensitive), Tr pool (25 drought-resistant + 25 drought-sensitive) and SBW pool (25 drought-resistant + 25 drought-sensitive), together with the parental materials, which were subsequently submitted to Lianchuan Biotechnology Co., Ltd., for BSA library construction and sequencing. The sequencing depth of the parents was 15 × , the average sequencing depth of each progeny mixed pool was 1 × , and the reference genome was the HAU-AD1_v1.1 version [20].

### qRT‒PCR analysis

The parents and individuals with extreme traits were planted in the cotton cultivation room and conventionally cultivated to the three-leaf stage, and 15% PEG_6000_ was used to simulate drought stress; the results at 0 h, 0.5 h, 1 h, 3 h, 6 h, 12 h, and 24 h after stress was induced were recorded. Leaf, root, and stem tissues were stored at ‒80°C. Total RNA was extracted according to the instructions of the polysaccharide and polyphenol total RNA extraction kit (Tiangen), and cDNA was synthesized by using the instructions of the reverse transcription 5X All-In-One RT MasterMix Kit (abm). According to the cDNA information of the genes, the NCBI Primer Tool was used to design fluorescent quantitative primers for the candidate genes (Table S1, Sheet 1). *GhUBQ7* was used as an internal reference gene, and the analysis of each sample was repeated three times. The ABI 7500 instrument was used to read the CT values of the internal reference primers and specific primers, and the gene expression was calculated according to the 2^–ΔΔCt^ method [21]. Excel 2010 was used for data statistics and arrangement, and GraphPad software was used for plotting.

### VIGS functional verification

The virus-induced gene silencing (VIGS) recipient material was planted in a cotton cultivation room and cultured routinely until the cotyledons unfolded. The CDSs of key genes were extracted from CottonFGD [22], and the genes were subsequently cloned using the cDNA of the parent material as a template. VIGS technology [23] was used to construct a silencing vector for key genes. After the leaves of cotton plants injected with pTRV2::*CLA* appeared albino, the pTRV2::00 and pTRV2::key gene young leaf tissue samples were subjected to RNA extraction and reverse transcription, after which the silencing efficiency of the key genes was measured via qRT‒PCR. Simultaneously, a simulated field drought stress treatment was carried out, the relevant phenotypes were photographed, and samples were taken at each timepoint and frozen in liquid nitrogen.

## Results

### Statistics and analysis of plant phenotypes

After drought stress treatment, the soil water content at different depths decreased by more than 15% compared with that before drought treatment, reaching the level observed under severe drought stress. Descriptive statistics were performed for the phenotypic traits, including agronomic, photosynthetic, biomass and yield indicators, of the RIL populations investigated under normal irrigation (CK) and drought stress (DS) conditions at three environmental points for two years (Table 1 and Table S1 sheet 2). The results showed that under the influence of drought stress, the phenotypic traits of the RIL populations all declined to varying degrees, and the coefficient of variation of the traits ranged from 7.18%-40.38%, which indicated that these traits were affected by drought stress to some extent. There is rich genetic diversity in these traits in the RIL population.

**Table 1:** Descriptive statistics of field traits of Shawan parents and populations in 2019

Traits	Treatment	Parents		Offspring			
		**Acala1517-08**	**XLZ36**	**Mean ± SD**	**CV%**	**max**	**min**
PH	CK	71.37	80.95	72.96 ± 7.43	10.18	90.45	56.15
	DS	59.05	66.10	67.52 ± 7.14	10.57	93.33	46.10
FBN	CK	9.83	9.93	9.81 ± 1.08	11.01	13.03	7.50
	DS	7.35	6.58	7.66 ± 0.81	10.57	10.80	5.50
EFBN	CK	8.13	7.63	7.22 ± 1.01	13.99	10.77	3.50
	DS	4.88	4.33	5.19 ± 0.83	15.99	9.30	3.10
BN	CK	10.07	9.68	8.77 ± 1.52	17.33	15.75	3.77
	DS	5.35	4.80	5.72 ± 1.07	18.71	10.80	3.40
EBN	CK	10.07	9.68	8.46 ± 1.48	17.49	15.50	3.17
	DS	4.18	4.80	5.16 ± 1.00	19.38	10.70	2.70
Pn	CK	26.60	29.97	26.08 ± 4.28	16.00	34.83	11.70
	DS	11.23	12.47	13.62 ± 3.61	27.00	23.80	5.70
Tr	CK	8.53	8.70	8.99 ± 1.57	17.00	13.60	5.10
	DS	9.43	6.83	8.04 ± 2.38	30.00	15.73	4.20
WUE	CK	3.10	3.43	3.01 ± 0.77	26.00	7.20	1.40
	DS	1.20	1.80	1.83 ± 0.66	36.00	8.77	0.57
Ci	CK	229.67	222.67	267.63 ± 37.85	14.00	358.33	178.00
	DS	295.67	243.67	236.57 ± 60.67	26.00	354.33	76.33
Gs	CK	456.00	499.00	644.78 ± 215.52	33.00	1383.00	171.33
	DS	439.33	237.67	333.56 ± 134.7	40.38	976.50	64.87
VPD	CK	2.40	2.27	2.03 ± 48.00	24.00	4.13	1.30
	DS	2.43	3.17	3.62 ± 1.26	35.00	6.57	1.30
SBW	CK	5.06	5.84	5.09 ± 0.73	14.34	6.50	1.67
	DS	4.98	5.05	4.85 ± 0.69	14.23	7.33	2.05
CSY	CK	101.17	116.73	101.77 ± 14.53	14.28	130.01	33.38
	DS	99.69	100.92	97.12 ± 13.8	14.21	146.64	41.00
CLY	CK	38.52	46.32	40.72 ± 6.87	16.87	55.35	9.81
	DS	39.79	44.60	39.68 ± 6.66	16.78	54.24	9.34
LP	CK	0.38	0.40	0.40 ± 0.03	7.50	0.45	0.29
	DS	0.40	0.44	0.40 ± 0.04	10.00	0.54	0.25
YPP	CK	50.18	57.38	43.12 ± 9.41	21.82	87.08	5.29
	DS	20.81	24.22	25.14 ± 6.29	25.02	59.10	6.88
Root	CK	23.73	23.17	18.94 ± 3.61	19.06	27.91	5.34
	DS	21.02	21.06	18.57 ± 3.82	20.57	38.52	10.06
Stem	CK	44.66	64.13	55.62 ± 12.83	23.07	114.11	32.24
	DS	45.14	57.02	54.11 ± 14.40	26.61	144.17	21.05
Leaf	CK	27.19	20.97	32.99 ± 8.27	25.07	74.76	8.46
	DS	24.97	32.19	33.05 ± 9.44	28.56	107.65	16.60
Boll	CK	61.19	71.57	78.07 ± 23.82	30.51	146.76	14.83
	DS	33.52	46.58	53.13 ± 14.98	28.19	101.22	19.19
OP	CK	133.04	156.66	166.69 ± 33.17	19.90	295.74	85.84
	DS	103.62	135.80	140.30 ± 31.94	22.77	331.50	77.95
RSR	CK	0.18	0.15	0.12 ± 0.02	16.67	0.20	0.07
	DS	0.21	0.16	0.14 ± 0.02	16.43	0.24	0.09

**Fig. 1:**
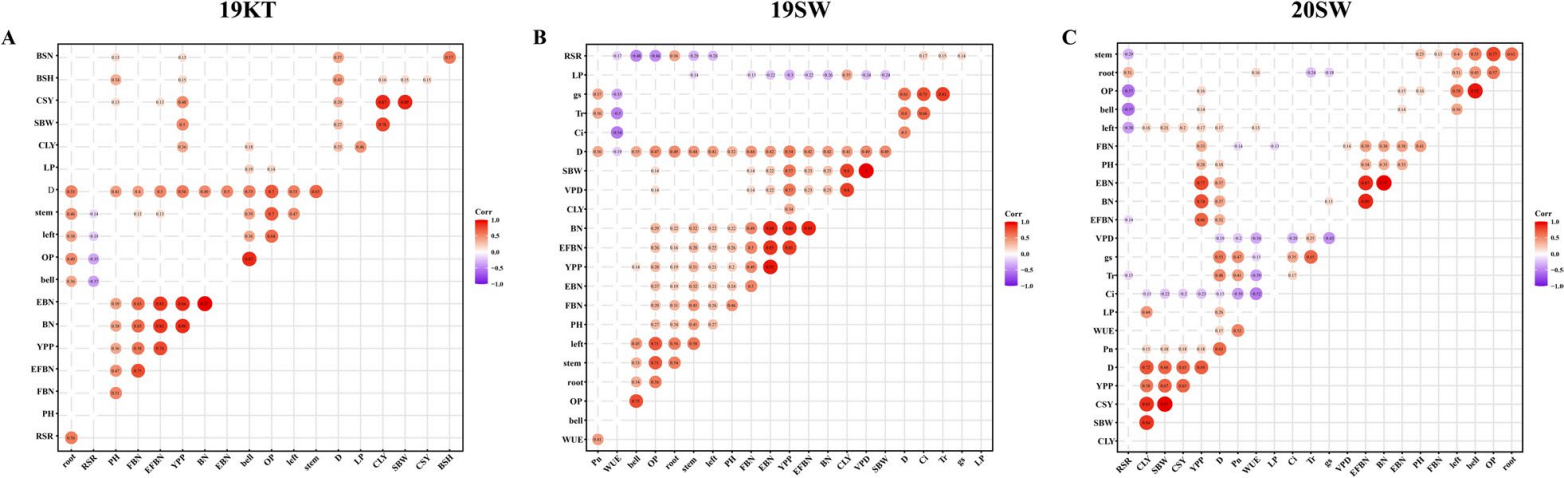
Statistics and analysis of the phenotypes of the three environmental points. **A**, **B**, and **C** show the correlations between the drought resistance coefficient DC and the comprehensive drought resistance measurement value of each trait index investigated at the 2019KT, 2019SW, and 2020SW points, respectively. The colors from red to blue represent positive to negative correlations

The correlation between key indicators and cotton drought resistance should be more significant. To study the relationship between the investigated traits and the drought resistance of cotton plants, we conducted a correlation analysis between the drought resistance coefficient (DC) and the comprehensive drought resistance measurement value (D) of the survey traits at each environmental point (Fig. 1A B C). The D values and the agronomic traits (PH, EFBN, and EBN), photosynthetic indicators (PN, Tr, Gs, and WUE), biomass [24] and yield traits (SBW, CLY, and YPP) were all strongly significantly positively correlated and were significantly negatively correlated with Ci, VPD and other traits, indicating that the above characteristics were closely related to the drought resistance of cotton. This indicated that drought had different effects on cotton morphology, photosynthesis, dry matter accumulation and yield formation and that there were relationships among the traits.

### Key indicators of drought resistance were selected by dimensionality reduction analysis

The complexity of cotton drought resistance-related traits led to correlation analysis revealing many drought resistance-related indicators. Principal component analysis revealed more critical drought resistance-related traits through dimensionality reduction. Principal component analysis (PCA) was used to reduce the dimensionality of 18, 21, and 22 traits surveyed in 2019KT, 2019SW, and 2020SW, respectively (Table S1 Sheet 3), and 6, 7, and 8 principal components were obtained, which together explained 82.30%, 83.39% and 85.75%, respectively, of the overall variation.

According to the PCA of the three environmental points, the principal component traits and their contribution rates obtained by dimensionality reduction were highly consistent. After the principal component factor loading matrix in each environment was sorted according to the size of the absolute value, multiple traits, such as YPP, Tr, SBW, PH, OP and RSR after drought stress, were screened out as key indicators for evaluating the drought resistance of cotton populations.

### Difference analysis of target traits in RIL population parents

Parental differences in target traits are the key to studying the overall characteristics of RIL populations. After drought stress, through statistical analysis of variance of the relative change rates of the traits of the parents at the three environmental points (Table S1 Sheet 4), it was found that at least at two environmental points, the relative change rates of the traits between the parents, including PH, SBW, EBN, Tr, WUE, Ci, and Gs, significantly differed. This finding is consistent with the index of the RIL population, which had a high contribution to the PCA and is a key trait of drought resistance and includes three traits: PH, SBW, and Tr. Therefore, in this study, PH, SBW, and Tr were selected as the key target traits for drought resistance. These traits are distributed within the categories of agronomic traits, yield indicators and photosynthetic indicators and respond to drought stress in cotton.

### Cluster analysis of the D value and D1 value to identify cotton drought resistance

**Fig. 2:**
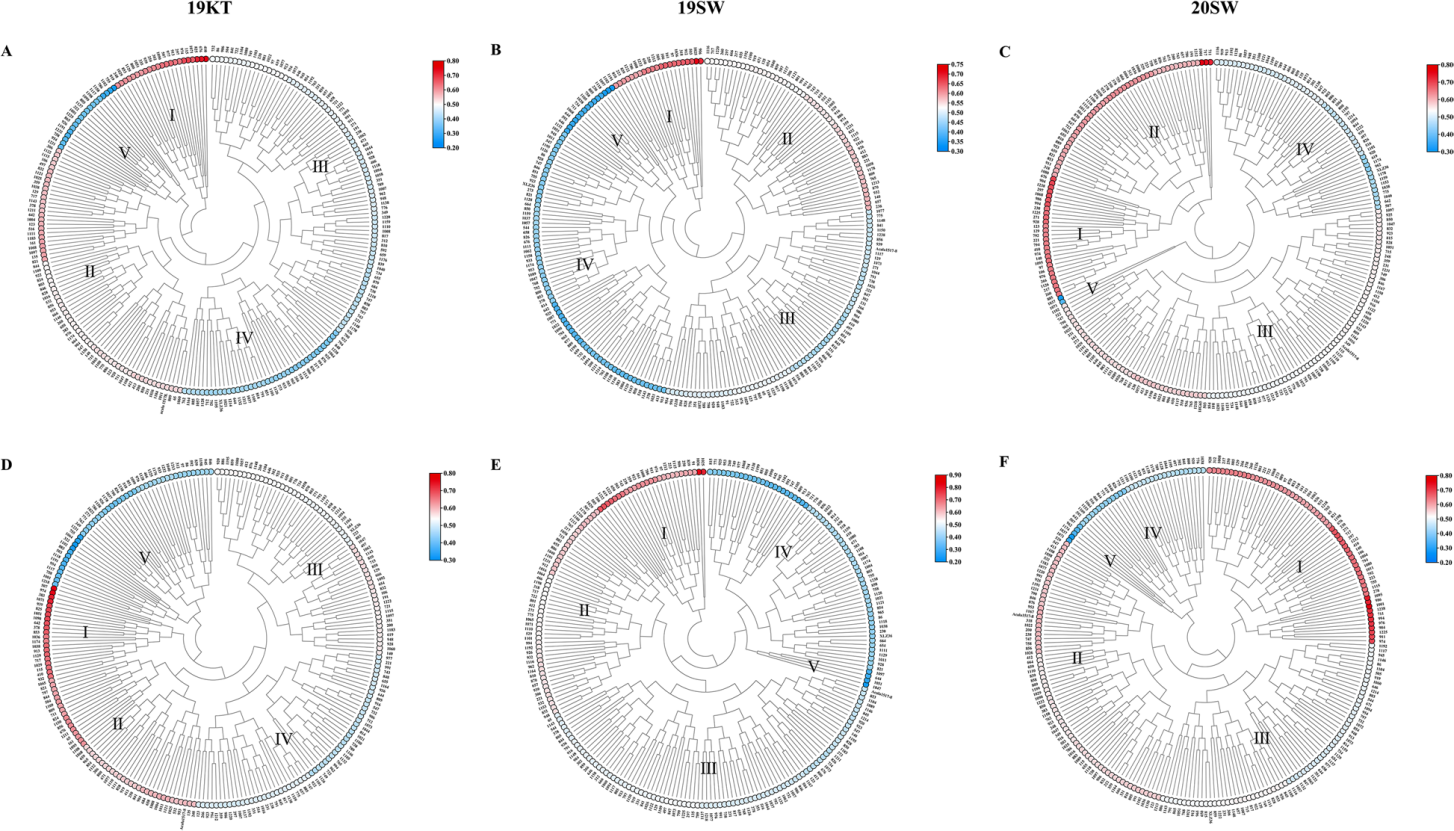
Cluster analysis chart of the D value and D1 value of 3 environmental points. **A** and **D**, **B** and **E**, and **C** and **F** are the results of cluster analysis of the D value and D1 value at the 2019KT, 2019SW, and 2020SW environmental points, respectively

To verify the reliability of the selection of the above drought resistance indicators, the DC values of the key traits (PH, SBW, and Tr) with stable performance and high contribution rates at multiple environmental points were used to obtain the D1 value, and the obtained D1 value and D value had different effects on the drought resistance of the population. Cluster analysis was performed, and the clustering results for D1 and D were roughly the same: Class I had strongly drought-resistant types; Class II had drought-resistant types; Class III had drought-tolerant types; Class IV had drought-sensitive types; and Class V had extremely drought-sensitive types (Fig. 2A B C D E F). Cultivars 1036, 97, 974, 911 and other materials have been identified as strong drought-resistant types in many environments and can be used as excellent breeding parents.

**Fig. 3:**
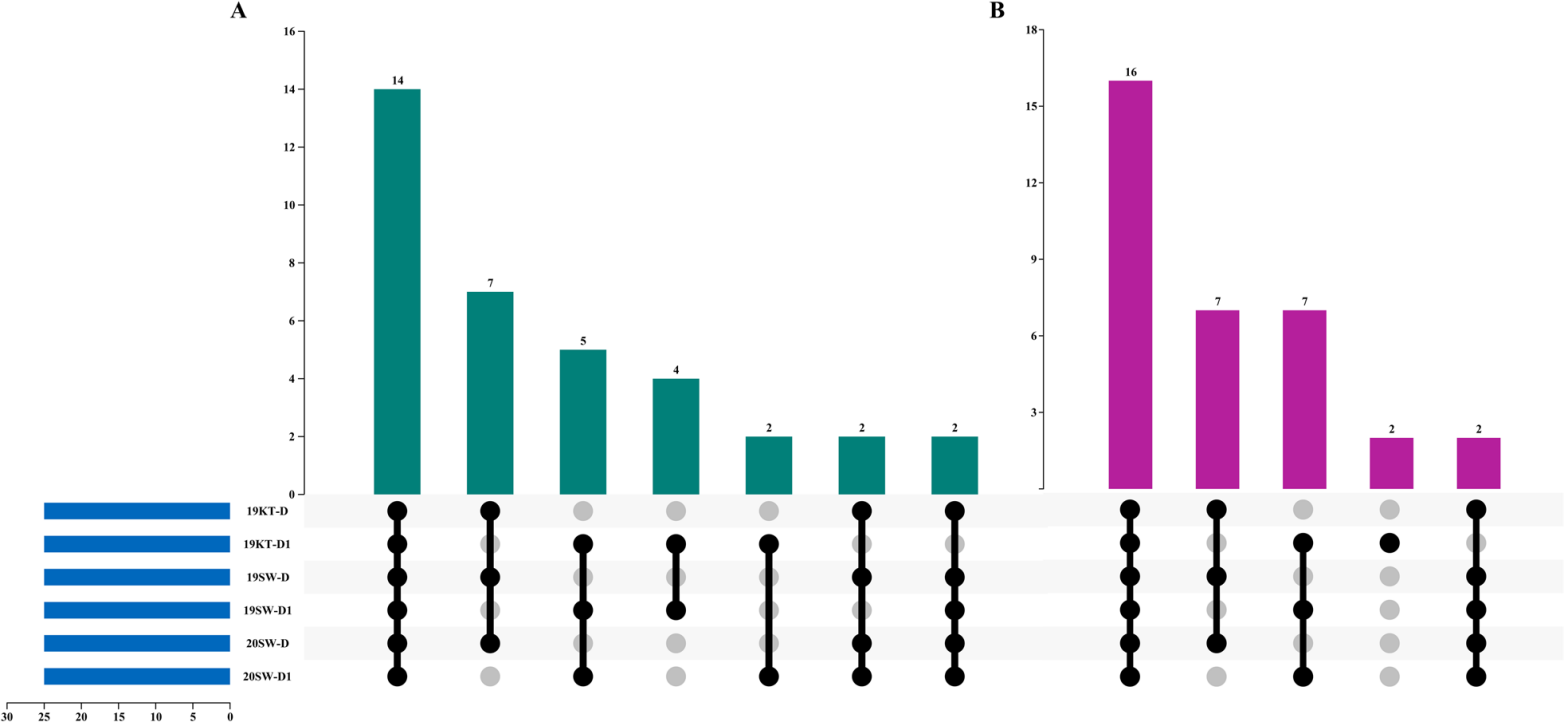
Consistency analysis of drought-resistant and drought-sensitive extreme materials at three environmental points. **A** and **B** show the results of intersection analysis of materials with extreme drought resistance and extreme drought sensitivity. The results were obtained by cluster analysis of the D value and D1 value at the 2019SW, 2019KT, and 2020SW environmental points

Furthermore, materials that accounted for the top 10% to 12.5% of the drought-resistant and drought-sensitive materials in the RIL population were considered extreme materials. Intersection analysis was performed on the materials screened by the two clustering methods (Fig. 3A B). The same materials accounted for 56%, 64%, and 72% of the drought-resistant extreme materials according to the D and D1 values at the 2019KT, 2019SW, and 2020SW environmental points, respectively. The drought-resistant extreme materials screened at all three environmental points accounted for 56% of the total materials; the drought-sensitive extreme materials that were screened accounted for 64%, 72%, and 72% of the total materials, and the drought-sensitive extreme materials screened at all three environmental points accounted for 64% of the total materials. These results all showed that the D1 values obtained for the key traits (PH, SBW, and Tr) were highly similar to the D values obtained for all the traits, and the evaluation results for the extreme materials were highly reliable. These findings further showed that the above traits are key components for evaluating the drought resistance index of cotton and can be used as key indices for evaluating the drought resistance of the population in this study.

### The establishment of an extreme mixing pool and the differences in extreme materials

**Fig. 4:**
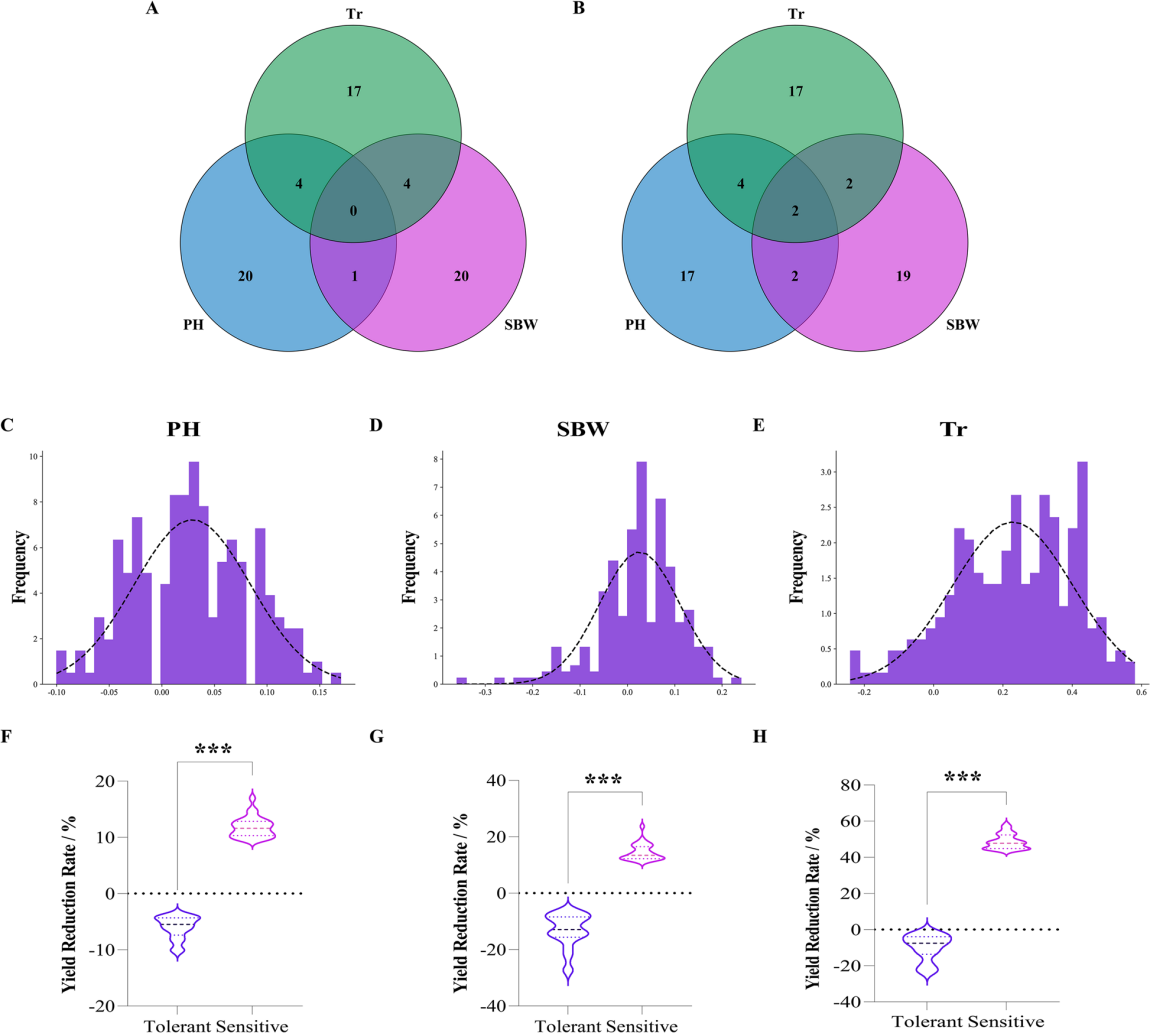
The establishment of the BSA sequencing pool and the differences in extreme materials. **A**. Material distribution among the PH, SBW, and Tr drought-resistant mixed pools. **B**. Material distribution among the PH-, SBW-, and Tr-sensitive dry mixed pools. **C**, **D**, and **E** are the normal distribution diagrams of the relative change rates of PH, SBW, and Tr in the RIL population. **F**, **G**, and **H** show the results of the T tests for the relative change rates of PH, SBW, and Tr in the extreme mixed pools. * represents *P* < 0.05, a significant difference; ** represents *P* < 0.01, a very significant difference; *** represents *P* < 0.001, an extremely significant difference

After obtaining the drought resistance results for the RIL population, an extreme mixed pool was constructed with PH, SBW, and Tr were the key indicators. Further analysis of the composition of the materials in each extreme mixed pool showed that 9 materials were found in drought-resistant mixed pools, with at least two traits in each drought-resistant mixed pool (Fig. 4A). Twelve materials were found in each drought-sensitive mixed pool, with at least two traits in the sensitive-dry mixed pools, and 2 materials were grouped in the sensitive-dry mixed pools for each trait (Fig. 4B).

In addition, the relative change rates of PH, SBW and Tr in the extreme materials were normally distributed, and the results showed that the change rates of PH, SBW and Tr after drought stress all conformed to a normal distribution (Fig. 4C D E). A t test (Fig. 4F G H) revealed that the relative change rate of PH ranged from -10.17 to 16.95, the relative change rate of SBW ranged from -27.76 to 23.70, the relative change rate of Tr ranged from -23.72 to 57.87, and the extreme relative change rates of the three traits were all highly significantly different.

### BSA-seq-located QTL intervals related to drought resistance

**Fig. 5:**
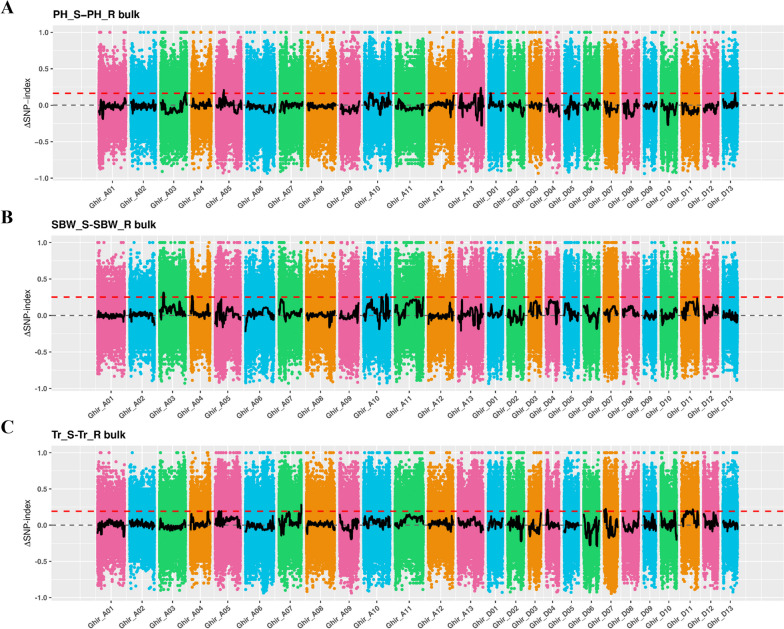
Manhattan plot of the PH, SBW and Tr values for the ΔSNP index. **A**, **B** and **C** are Manhattan plots of QTL positioning based on the SNP-index method for the PH, SBW and Tr extreme mixing pools, respectively

The parental Acala1517-08, XLZ36, PH pool, SBW pool, and Tr pool were analyzed via BSA-seq, and the relevant QTL intervals were mapped via the Euclidean distance (ED) association method and the SNP-index method. Sliding window calculations were performed according to the positioning results, and the intersection area of the top 1% of the SNP index values and the top 1% of the ED values was selected as the key research interval. Finally, the mapping results based on the PH, SBW and Tr traits showed that when the ΔSNP index was above quantile 99, multiple QTL intervals were still located on multiple chromosomes. Based on the positioning interval of each trait, in this study, an interval with a higher ΔSNP index was selected as the focus of research. A total of 6.27 Mb of major drought resistance QTL intervals were selected on chromosomes A13 (3.2 Mb), A10 (2.45 Mb) and A07 (0.62 Mb), containing a total of 248 genes (Fig. 5A B C). Although there were repeated materials in the three groups of antisensitive pools, the mapping results did not overlap within the same segment. Only PH and SBW were also located on the A10 chromosome and at relatively close positions. Moreover, in subsequent GWAS research conducted by the research team, SBW was also found to be located on the same A10 chromosome, which is an important reason for choosing this positioning interval in this study. This finding may be related to the fact that cotton drought resistance is controlled by microeffect polygenes rather than specific chromosomes or genes that can determine drought resistance.

### Screening of candidate genes

**Fig. 6:**
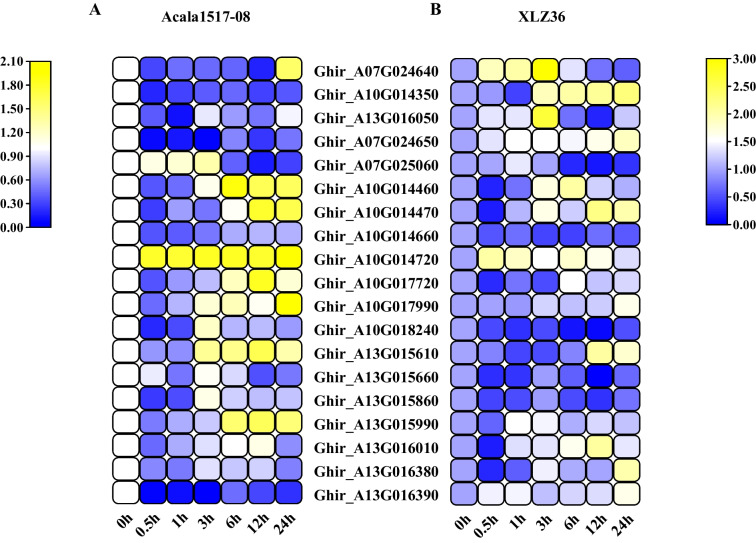
Expression of 19 candidate genes under drought stress. A. qRT‒PCR analysis of 19 candidate genes in Acala1517-08; B. qRT‒PCR analysis of 19 candidate genes in XLZ36. The expression level was normalized to -log2 (expression value + 1)

Based on the annotation results of the candidate genes and the polymorphic SNP information between parents, 19 candidate genes that may respond to drought stress were selected from 248 candidate genes. In addition, the expression patterns of these genes after drought stress were analyzed by qRT‒PCR (Fig. 6A B). After data normalization, three genes, *Ghir_A13G016050* (*GhF6'H1*), *Ghir_A10G014350* (*Gh3AT1*), and *Ghir_A07G024640* (*GhPER55*), were shown to have opposite differential high expression between the two parents during different stress periods, and it was speculated that these genes may be involved in the drought stress response.

### qRT‒PCR analysis of the drought stress response of three key genes

**Fig. 7:**
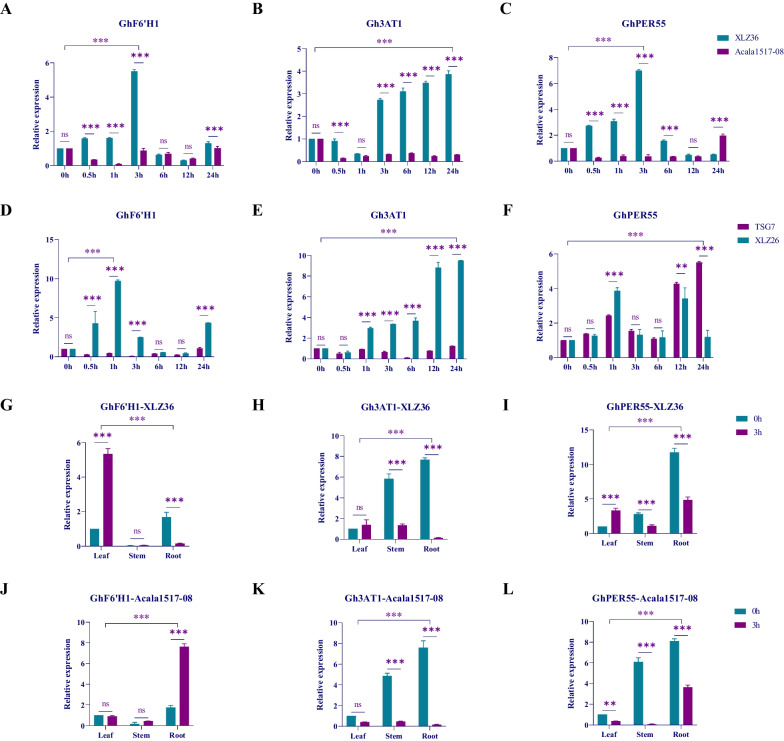
qRT‒PCR and tissue-specific analysis of 3 key genes. **A** and **D** show the expression levels of GhF6'H1 in parental and extreme drought stress conditions; **B** and **E** show the expression levels of Gh3AT1 in parental and extreme drought stress conditions; **C** and **F** show the expression levels of GhPER55 in parental and extreme drought stress conditions; **G** and **J** show the tissue specificity of GhF6'H1 in parental drought stress conditions; **H** and **K** show the expression of Gh3AT1 in parental drought stress conditions; and **I** and **L** show the tissue specificity of GhPER55 in parental drought stress conditions. The statistical method used was two-way analysis of variance; * represents *P* < 0.05, a significant difference; ** represents *P* < 0.01, a very significant difference; and *** represents *P* < 0.001, an extremely significant difference

The group parents represent the characteristics of the group, and the main germplasm materials represent the universality of the characteristics. The drought-resistant material TSG7 and the drought-sensitive material XLZ26 were subjected to qRT‒PCR analysis to further verify the changes in the expression of the three key genes after drought stress in other extreme materials. The results showed that the expression of *GhF6'H1* was upregulated within 1 h of drought stress in the drought-sensitive materials XLZ36 and XLZ26 and then gradually decreased and showed a fluctuating decrease in expression or no expression in the drought-resistant materials (Fig. 7A D); The drought-sensitive materials XLZ36 and XLZ26 were highly expression of *Gh3AT1* within 0–24 h, with increasing expression with increasing stress duration (Fig. 7B E); The expression of *GhPER55* in the drought-sensitive materials XLZ36 and XLZ26 first increased and then decreased. The expression trend of *GhPER55* in the drought-resistant material TSG7 was different from that in the drought-resistant parent material Acala1517-08, fluctuating between highly expressed up- and downregulated expression (Fig. 7C F). These results showed that the responses of the three key genes were more significant in the drought-sensitive materials than in the control materials, and it was speculated that these genes might be involved in negative regulatory responses.

Furthermore, to study the participation of the three key genes in the response to drought in different tissues of cotton, the roots, stems, and leaves at 3 h, which exhibited extremely significant differential expression between the two parents, were specifically selected, and the tissue specificity of the three key genes was analyzed (Fig. 7). After drought stress, the expression of *GhF6'H1* increased significantly in the leaf tissue of the drought-sensitive parent, and *GhF6'H1* was highly expressed in the root tissue of the drought-resistant parent (Fig. 7G J); under the influence of drought stress, the expression of *Gh3AT1* slightly increased in the leaf tissue of the drought-sensitive parent LXZ36 and was highly expressed in the root tissue of the drought-resistant parent. In the drought treatment, both parental materials presented high expression in the roots (Fig. 7H K); Similarly, the expression of *GhPER55* significantly increased in the leaf tissue of the drought-sensitive parent but slightly decreased in that of the drought-resistant parent, and the expression of both the root and stem tissues significantly decreased in both parents. We speculated that *GhPER55* may play a role in the response of leaf tissue to drought stress (Fig. 7I L).

### Cotton VIGS experiment verification of the functions of key genes

**Fig. 8:**
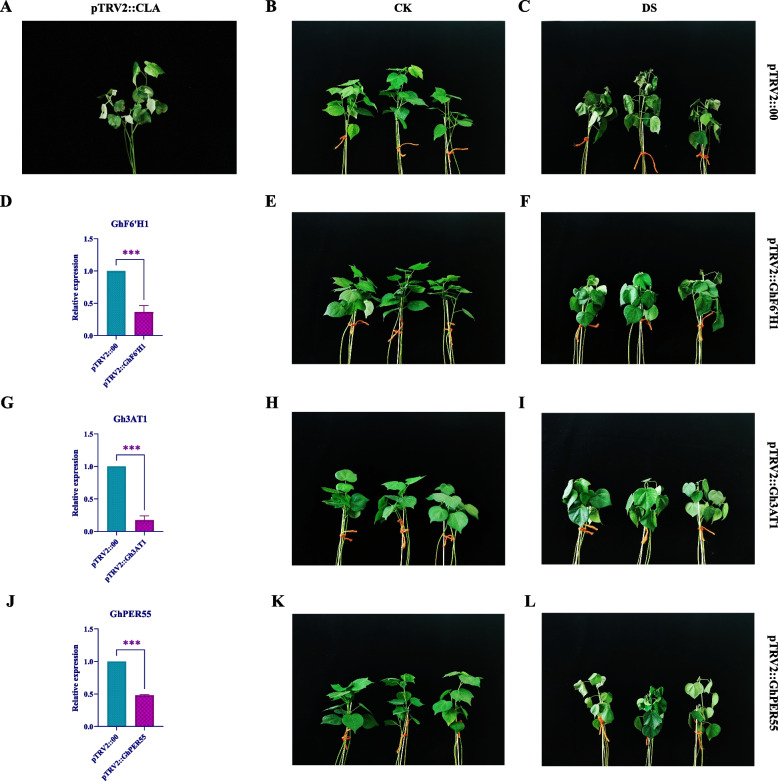
VIGS of 3 key genes. **A** shows the albino phenotype of pTRV2::CLA plants; **D**, **G**, and **J** show the silencing efficiency of pTRV2::GhF6'H1, pTRV2::Gh3AT1 and pTRV2::GhPER55 plants; **B** and **C** show the normal pTRV2::00 plant phenotypic changes after the control (CK) and drought stress (DS) treatments; **E** and **F** show the phenotypic changes in pTRV2::GhF6'H1 plants after the control treatment (CK) and drought stress treatment (DS); **H** and **I** show the phenotypic changes in pTRV2::Gh3AT1 plants after the control treatment (CK) and drought stress treatment (DS); and **K** and **L** show the phenotypes of pTRV2::GhPER55 plants after the control treatment (CK) and drought stress (DS), respectively. Two-way analysis of variance was used for statistical analysis. * represents *P* < 0.05, a significant difference; ** represents *P* < 0.01, a highly significant difference; *** represents *P* < 0.001, an extremely significant difference

VIGS is an extremely important method for gene function analysis [25]. To verify whether the three key genes are involved in the drought stress response of cotton, we carried out cotton silencing experiments on the three genes in the drought-sensitive parent material XLZ36. After the leaves of the pTRV2::*CLA* cotton plants exhibited an albino phenotype (Fig. 8A), multiple pTRV2::00 plants and 3 gene-silenced plants were randomly selected to determine the gene silencing efficiency. The results showed (Fig. 8D G J) that the silencing experiment was relatively successful, ensuring the accuracy of the subsequent experiments. After silencing, the phenotype was observed, and it was found that the leaves of the pTRV2::00 plants wilted severely after being subjected to drought stress (Fig. 8B C); the leaves of the pTRV2::*GhF6'H1* and pTRV2::*Gh3AT1* plants wilted slightly (Fig. 8E F H I); and the leaves of the pTRV2::*GhPER55* plants showed severe wilting, which was less severe than that of the pTRV2::00 plants (Fig. 8K L). Overall, the silencing of the three key genes increased the drought resistance of the drought-sensitive parent XLZ36 to a certain extent after being subjected to drought stress.

## Discussion

The drought resistance of plants is a quantitative trait controlled by polygenes with microeffects, and it is more scientific and reasonable to comprehensively evaluate the drought resistance of plants with multidimensional indicators [26]. Based on the drought resistance identification of large populations, complex phenotypes increase the difficulty of investigations, and it is very important to focus on key phenotypes. In bilberry plants [27], based on statistical methods such as principal component analysis (PCA) and stepwise linear regression, 16 phenotypic and physiological indicators related to pH resistance were attributed to 7 indicators, including plant height, SOD activity and SPAD. In wheat, 12 physiological and biochemical indicators, including stomatal conductance, proline and malondialdehyde, were found to be associated with drought resistance via the use of multivariate statistical methods. In plum [28], through cluster analysis and principal component analysis, multiple traits related to drought resistance were attributed to five indicators, leaf area, stomatal density, and stomatal area, to evaluate drought resistance. Similar methods have also been applied in plants such as melon [29], grape [30], and canna [31]. In this study, through a series of multivariate statistical analyses, such as principal component dimensionality reduction analysis combined with parental relative change rate analysis of variance, the key indicators of the 18, 21, and 22 traits investigated at the three environmental points were the focus, and PH, SBW and Tr were determined to be the three key indicators for evaluating the drought resistance of the RIL populations.

PH, SBW and Tr, key traits, have been investigated as the main traits involved in the identification of drought resistance and related QTL mapping in cotton. Abdelraheem et al. [32] evaluated the drought resistance of RIL populations based on multiple traits related to morphology, physiology, yield, and fiber quality, including PH and SBW, under drought conditions in greenhouses and fields and used 1004 DNA polymorphisms. A genetic map was constructed from the marker loci, and 165 QTL related to drought and salt tolerance were detected on multiple chromosomes. Zhang et al. [9] evaluated the high-temperature resistance of backcrossed inbred lines (BILs) by using traits such as plant height (PH) and fresh plum weight (SW) under control and PEG treatment conditions at room temperature and identified 6 QTL regions associated with cotton located on chromosomes A10, D11, etc. Li et al. [12] used 18 trait indicators, such as PH and SBW, to identify 517 upland cotton natural population materials for drought resistance identification and associated gene mapping analysis and found 39 QTLs related to drought resistance. Based on our previous research results [13], five indicators, namely, plant height (PH), effective branch number (EFBN), single boll weight (SBW), transpiration rate (Tr) and chlorophyll (ChI), were defined as the traits associated with drought resistance in cotton. By revealing the key indicators associated with sex identification and evaluating drought resistance in genetic and natural populations, this study also provides an extension of previous research results, further focusing on key indicators. Furthermore, these results showed that some of these mapped QTLs exist on the same chromosome as the QTLs mapped in this study, but QTLs in the same interval have not yet been reported. These findings provide additional information related to the QTLs associated with drought resistance in cotton and indicate that the three selected key genes may constitute important high-quality genetic resources.

*GhF6'H1* belongs to the feruloyl CoA ortho-hydroxylase 1 family, and the members of this family play important roles in the growth, development and stress resistance of plants. In *Arabidopsis*, *F6'H1* is a key step in catalyzing the biosynthesis of the coumarin scopoletin, which has a direct impact on the processing of lignocellulosic biomass [33, 34]. In addition, scopoletin, a secondary metabolite, has a series of important functions in plants. In *Sophora davidii* [35], scopoletin is involved in phenylpropanoid biosynthesis and flavonoid biosynthesis, and these pathways affect many important plant traits and stress responses [36, 37]. In soybean [38], scopoletin can inhibit the formation of rust pathogen preinfection structures and penetration into plants. In *Arabidopsis* [39], scopoletin selectively affects the assembly of rhizosphere microbial communities. In the present study, this gene was discovered within the QTL interval mapped to the PH trait under drought stress, which showed that GhF6'H1 may be related to the growth and development of cotton plants under drought stress conditions.

Gh3AT1 belongs to a class of acyl-CoA-dominated coumaryl transferases that play multiple roles in the modification of anthocyanins, flavonoids, and volatile esters in plant secondary metabolism [40]. However, studies on the drought resistance of plants, especially cotton plants, are rare. In this study, this gene was discovered in the QTL interval mapped for SBW traits under drought stress. Although there is currently no research progress in this area, Gh3AT1 may be important for cotton fiber development under stress.

GhPER55 belongs to the peroxidase family and is involved mainly in endogenous reactive oxygen species scavenging, lignin synthesis and degradation, and the stress response to environmental stress [41, 42]. In the present study, this gene was detected within the QTL interval mapped to the Tr trait under drought stress, which may indicate that GhPER55 is closely related to the transpiration rate and stomatal size of cotton plants after drought stress. Moreover, this study also conducted preliminary functional verification of these three key genes. qRT‒PCR and VIGS analyses of the phenotypic changes revealed that these genes are involved in the drought stress response in cotton, but the specific regulatory mechanisms involved require further study.

## Conclusions

From the perspective of genetic improvement, in this study, the drought resistance of RIL populations of upland cotton was identified, and statistical methods were used to focus on key traits, namely, PH, SBW, and Tr, from many drought-related traits. Based on these three key traits, the drought resistance of RIL populations was evaluated and excellent parent materials were screened, and the results were highly consistent with the drought resistance evaluation results for multiple traits and multiple environments. Furthermore, key QTL intervals were located based on the key traits, and three key genes involved in the drought stress response were selected and preliminarily verified. These results provide relevant data for the further improvement of cotton drought resistance identification systems and highlight high-quality candidate genes for cotton molecular breeding projects.

### Supplementary Information

**Additional file 1.** Supplementary tables

## Data Availability

The data related to simplified BSA-seq genome resequencing are available in the NCBI Sequence Read Archive database under accession number PRJNA1024065. All the other datasets supporting the results of this article are included in this article and its attached files.

## Abbreviations

QTL: Quantitative trait locus
BSA: Bulked segregant analysis
RIL: Recombinant inbred line
PH: Plant height
SBW: Single boll weight
Tr: Transpiration rate
qRT‒PCR: Quantitative real-time polymerase chain reaction
VIGS: Virus-induced gene silencing
